# Asymmetric Upper Limb Dysfunction Demonstrated at the Bedside: A Phenomenology Video

**DOI:** 10.1002/mdc3.70624

**Published:** 2026-04-03

**Authors:** Gero Lueg, Martin Fruth, Nina Rosa Neuendorff, Rainer Wirth, Markus A. Hobert

**Affiliations:** ^1^ Department of Geriatric Medicine, Marien Hospital Herne Ruhr University Bochum Herne Germany; ^2^ Department of Diagnostic, Interventional Radiology and Nuclear Medicine, Marien Hospital Herne Ruhr University Bochum Herne Germany; ^3^ Department of Neurology, University Medical Center Schleswig‐Holstein Campus Lübeck and University of Lübeck Lübeck Germany

## Question

A 70‐year‐old woman presented with recurrent falls over 1 year. Neurological examination revealed vertical gaze palsy, left‐predominant akinetic‐rigid parkinsonism, and postural instability. Brain magnetic resonance imaging showed midbrain atrophy.

During the paper‐toss test (PTT),^1^ the patient was asked to throw a balled‐up piece of paper with each hand. The right hand performed normally. When using the left (non‐dominant) hand, the patient initiated the throwing movement appropriately but failed to release the paper, resulting in an interrupted throwing motion (Video 1).

**Video 1:** During the paper‐toss test, the patient performs the task normally with the right hand. When using the left hand, she initiates the throwing movement but fails to release the paper ball.

What Abnormality Is Demonstrated in this Video?

1. Alien limb phenomenon
2. Unilateral limb apraxia with release failure
3. Motor perseveration
4. Bradykinesia

## Answer

Correct answer: Unilateral limb apraxia with release failure.

This video demonstrates a unilateral disturbance of a learned, goal‐directed upper‐limb action, showing failure to complete a voluntarily initiated motor sequence with the affected hand. During the PTT, the action breaks down at the release phase, resulting in a fragmented motor sequence.

The abnormality shown is not an alien limb phenomenon, which is characterized by autonomous, unintended limb activity independent of voluntary motor intent. No such behavior is present. Mild dystonic posturing may coexist but does not fully explain the selective failure to release the object. The preserved initiation of movement and selective breakdown at release favor a praxis disorder over bradykinesia or simple weakness.

Corticobasal syndrome (CBS) comprises multiple, often co‐occurring but dissociable deficits, including apraxia, dystonia, and alien limb phenomena. The PTT probes a complex lateralized praxis disorder affecting learned hand and finger action sequences.^2^

The PTT provides a simple bedside maneuver in which impaired release of the paper ball serves as a marker of lateralized upper‐limb praxis dysfunction. The overall pattern was most consistent with progressive supranuclear palsy presenting with CBS.

## Author Roles

(1) Research project: A. Conception, B. Organization, C. Execution; (2) Manuscript Preparation: A. Writing of the First Draft, B. Review and Critique.

G.L.: 1A, 1B, 1C, 2A

M.F.: 1C, 2B

N.R.N.: 1B, 2B

R.W.: 1B, 2B

M.A.H.: 1A, 2B

## Disclosures

**Ethical compliance statement:** The retrospective study protocol including the present patient was reviewed and approved by the Ethics Committee of Westphalia‐Lippe (reference number 2025‐148‐f‐S). Written informed consent for participation and for video recording and publication of the video material was obtained from the patient. We confirm that we have read the Journal's position on issues involved in ethical publication and affirm that this work is consistent with those guidelines.

**Funding and Conflicts of Interest:** No specific funding was provided for this study. The authors declare that there are no relevant conflicts of interest.

**Financial disclosures in the past 12 months:** The authors declare that no other financial disclosures are required.

## Data Availability

The data that support the findings of this study are available on request from the corresponding author. The data are not publicly available due to privacy or ethical restrictions.
